# Making Invisible RNA Visible: Discriminative Sequencing Methods for RNA Molecules with Specific Terminal Formations

**DOI:** 10.3390/biom12050611

**Published:** 2022-04-20

**Authors:** Megumi Shigematsu, Yohei Kirino

**Affiliations:** Computational Medicine Center, Sidney Kimmel Medical College, Thomas Jefferson University, Philadelphia, PA 19107, USA; megumi.shigematsu@jefferson.edu

**Keywords:** short non-coding RNA, RNA-seq, 2′,3′-cyclic phosphate, cP-RNA-seq, phospho-seq, 5’-hydroxyl-seq

## Abstract

Next generation sequencing of RNA molecules (RNA-seq) has become a common tool to characterize the expression profiles of RNAs and their regulations in normal physiological processes and diseases. Although increasingly accumulating RNA-seq data are widely available through publicly accessible sites, most of the data for short non-coding RNAs (sncRNAs) have been obtained for microRNA (miRNA) analyses by standard RNA-seq, which only capture the sncRNAs with 5′-phosphate (5′-P) and 3′-hydroxyl (3′-OH) ends. The sncRNAs with other terminal formations such as those with a 5′-hydroxyl end (5′-OH), a 3′-phosphate (3′-P) end, or a 2′,3′-cyclic phosphate end (2′,3′-cP) cannot be efficiently amplified and sequenced by standard RNA-seq. Due to the invisibility in standard RNA-seq data, these non-miRNA-sncRNAs have been a hidden component in the transcriptome. However, as the functional significances of these sncRNAs have become increasingly apparent, specific RNA-seq methods compatible with various terminal formations of sncRNAs have been developed and started shedding light on the previously unrecognized sncRNAs that lack 5′-P/3′-OH ends. In this review, we summarize the expanding world of sncRNAs with various terminal formations and the strategic approaches of specific RNA-seq methods to distinctively characterize their expression profiles.

## 1. Introduction

Next generation sequencing of RNA molecules (RNA-seq) has revolutionized transcriptome analyses and has become a common tool to identify RNA expression profiles. Increasingly, accumulating RNA-seq data have become widely available through publicly accessible sites, such as the National Center for Biotechnology Information (NCBI)’s Sequence Read Archive (SRA), not only for basic molecular, cellular, and computational biology research, but also for broader application with clinical contexts. In addition to studies from individual research groups, consortiums of large-scale transcriptome projects have been collecting numerous RNA-seq datasets. For example, The Cancer Genome Atlas (TCGA), aiming to compile cancer-associated genetic/epigenetic information, encompasses a total of 43 “microRNA (miRNA)-seq” projects, which have generated 461,071 RNA-seq datasets from 17,866 cases (https://www.cancer.gov/tcga (accessed on 15 March 2022)). These datasets can be redirected from various sites such as the cBioPortal for Cancer Genomics [1,2] and Catalogue Of Somatic Mutations In Cancer (COSMIC) [3].

Given a wide variety of the lengths and properties of cellular RNA molecules, no single RNA-seq method can capture all cellular RNA species expressed. Consequently, various RNA-seq methods have been developed for specifically targeted RNA molecules. This review focuses on short non-coding RNAs (sncRNAs) whose lengths are shorter than mature transfer RNA (tRNA) molecules (less than ~60 nucleotides (nt)). Most previous studies targeting the sncRNAs, such as those in TCGA project, have focused on miRNAs by using standard small RNA-seq method. However, it has become increasingly apparent that not only miRNAs but also other types of sncRNAs are abundantly expressed as functional molecules. Many of these sncRNAs cannot be efficiently captured by standard small RNA-seq mainly due to the method’s incompatibility with their terminal structures, thus underrepresenting the non-miRNA-sncRNAs in many of the current transcriptome analyses.

**Figure 1:**
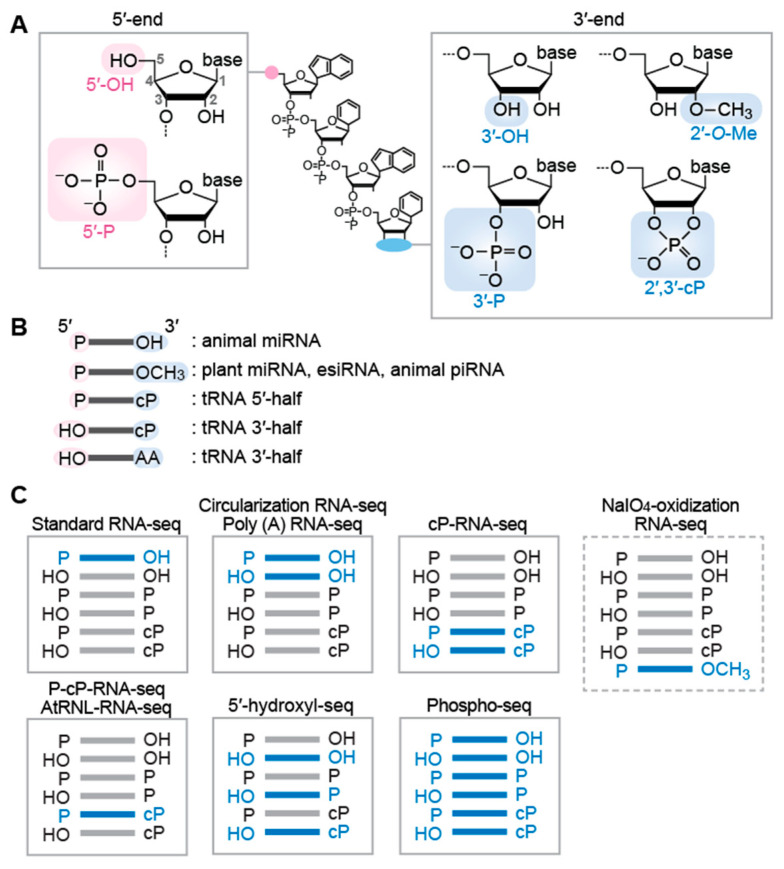
Terminal formations of sncRNAs and their compatible sequencings: (**A**) Chemical structures of RNA termini. (**B**) Terminal formations of sncRNAs. (**C**) Targeted sncRNAs (shown in blue) in each RNA-seq methods.

Cellular sncRNA molecules generally possess either a hydroxyl group (OH), a monophosphate (P), or a 2′,3′-cyclic phosphate (2′,3′-cP) at their termini (Figure 1A). The 2′,3′-cP end is formed only at the 3′-end of RNAs, in which the 2′- and 3′-positions of ribose is bridged by the phosphate. These terminal states of each sncRNA are determined by catalytic machineries of RNA cleavage that produces the sncRNAs. In many cases, the terminal formations are not just consequences of the biogenesis mechanism, but are critical in the stability and function of the sncRNAs. In addition to terminal phosphate states, terminal post-transcriptional modifications, such as 2′-*O*-methyl group (2′-*O*-Me) at the 3′-terminal ribose, further differentiate terminal forms of RNA molecules (Figure 1A). The current standard RNA-seq for sncRNAs relies on 5′-P/3′-OH ends of RNAs, which makes the RNAs with other terminal structures invisible in the RNA-seq data. While those RNAs have been forming a hidden component in most sncRNA transcriptome analyses, recent developments of specific RNA-seq compatible with various terminal structures have started shedding light on previously unrecognized sncRNAs that lack the 5′-P/3′-OH ends. In this review, we summarize the expanding world of sncRNAs with various terminal structures and list RNA-seq methods to reveal their expression profiles that have been uncaptured by standard small RNA-seq methods.

## 2. Expanding World of sncRNAs and Their 5′- and 3′-Ends

The first distinct class of functional sncRNAs was discovered in the studies of *C. elegans* [4,5], followed by the discovery of similar sncRNAs in other organisms [6]. While these 19–24-nt sncRNAs, now known as miRNAs, have become the best-characterized type of sncRNAs, continuous exploration and characterization thus far have identified further functional sncRNAs with distinct properties, biogenesis pathways, terminal formations, and molecular functions.

### 2.1. miRNA and esiRNA

miRNAs are bound by Argonaute (AGO) proteins to form RNA-Induced Silencing Complex (RISC) and act as guide molecules to target complementary regions of messenger RNAs (mRNAs) and other ncRNAs, silencing their expression through translational repression and/or promotion of RNA decay [6,7]. miRNAs are estimated to regulate the expression of almost all mRNAs and a wide variety of ncRNAs, crucially impacting normal developmental and physiological processes and diseases [8,9]. In the canonical pathway of miRNA biogenesis, a long hairpin-shaped primary miRNA (pri-miRNA) is first cleaved by Drosha, a member of Ribonuclease (RNase) III, generating a shortened hairpin-shaped precursor miRNA (pre-miRNA) harboring the 5′-P and 3′-OH ends [6,10,11,12]. The pre-miRNA is further cleaved by Dicer, another member of RNase III, leaving the 5′-P and 3′-OH ends in mature miRNA molecules [6,10,11,12] (Figure 1B). While Dicer-mediated cleavage is the final canonical biogenesis step for animal miRNAs, plant miRNAs further undergo a 3′-terminal methylation step mediated by HUA ENHANCER 1 (HEN1) methyltransferase, forming the 2′-*O*-Me end [13,14] (Figure 1B).

In addition to miRNAs, animals express endogenous small interfering RNAs (esiRNAs), a similar yet distinct type of sncRNA. esiRNAs are also bound by AGO proteins and regulate transcription from transposons or post-transcriptionally silence gene expression [15,16]. esiRNAs are produced from double-stranded RNAs (dsRNAs) or long step-loop RNAs by Dicer-mediated cleavage, followed by HEN1-catalyzed 3′-terminal methylation, resulting in their 5′-P and 2′-*O*-Me end [15,16,17,18] (Figure 1B).

### 2.2. piRNA

Argonaute family proteins are divided into AGO and PIWI subclades. While AGO subclades bind to miRNAs and esiRNAs, PIWI subclades bind to a distinct class of longer (23–34-nt) sncRNAs, termed PIWI-interacting RNAs (piRNAs) [19,20]. Unlike miRNAs that are ubiquitously expressed in all cells/tissues, PIWI proteins, and their bound piRNAs are predominantly expressed in animal germlines, where they maintain genome integrity by silencing transposable elements and regulating the expression of other targets [19,20]. The 5′-end of mature piRNAs is formed by Zucchini/mitoPLD endonuclease [21,22] or PIWI/piRNA complexes via a ping-pong amplification loop [23,24], while the 3′-end is generated from exonucleolytic cleavage by a Trimmer/PNLDC1 [25], followed by HEN1-mediated methylation [26,27,28]. Consequently, mature piRNAs possess 5′-P and 2′-*O*-Me ends [29,30] (Figure 1B).

### 2.3. tRNA-Derived sncRNA: tRNA Half and tRF

Although tRNAs have been best known as adapter components of translational machinery, tRNAs are now further known as a source of functional sncRNAs [31,32,33,34]. In many organisms, specific tRNA-derived sncRNAs are generated from mature tRNAs and their precursor transcripts, not as random degradation products but as functional molecules. The expression of tRNA-derived sncRNAs does not usually affect mature tRNA pools, but the sncRNAs themselves are involved in various biological processes beyond translation. tRNA-derived sncRNAs are classified into two groups: tRNA halves and tRNA-derived fragments (tRFs) [31,32,33,34].

tRNA halves are 30–45-nt in length, derived from either the 5′- or 3′-portion of mature tRNAs, and produced by endonucleolytic cleavage of tRNA anticodon-loop. In mammalian cells, angiogenin (ANG), a member of the RNase A superfamily, is the enzyme responsible for the anticodon cleavage [35,36]. ANG-mediated tRNA cleavage can be induced by various biological factors/phenomena such as stress stimuli [35,36], sex hormone signaling pathways [37], and immune response upon mycobacterial infection [38,39]. The generated tRNA halves have been shown to exhibit a variety of molecular functions. Stress-induced tRNA halves promote stress granule formation and regulate translation [35,40,41,42], while sex hormone-induced tRNA halves promote proliferation of hormone-dependent cancer cells [37]. Infection-induced tRNA halves could function as immune boosters by activating endosomal Toll-like receptor 7 [38]. tRNA halves can further serve as direct precursors for shorter sncRNAs such as piRNAs [43,44]. Because ANG-medicated cleavage leaves a 2′,3′-cP and 5′-P in 5′- and 3′-cleavage products [45], respectively, 5′-tRNA halves contain a 5′-P (from mature tRNAs) and a 2′,3′-cP, while 3′-tRNA halves possess a 5′-OH and an amino acid (AA) at the 3′-end (from mature tRNAs) (Figure 1B), which has been experimentally validated [37]. Regarding 3′-tRNA halves, cP-specific sequencing (see below) identified abundant 3′-tRNA halves lacking 3′-terminal adenosine at nucleotide position (np) 76 of tRNA, further indicating the presence of 3′-tRNA halves with the 5′-OH/2′,3′-cP ends [46,47] (Figure 1B).

tRFs are generally shorter than tRNA halves and comprise the rest of tRNA-derived sncRNAs. tRFs can mainly be subclassified into 5′-tRFs, 3′-tRFs, and i-tRFs [31,32,33,34]. While 5′- and 3′-tRFs correspond to the 5′- and 3′-parts of mature tRNAs containing processed 5′-P and 3′-AA termini, respectively; i-tRFs are derived wholly from internal parts of mature tRNAs. These tRFs are defined as the fragments generated by cleavages anywhere within mature tRNAs except for anticodon-loop. Although Dicer and ANG are involved in the biogenesis of some tRFs [48,49], detailed regulatory mechanisms and other biogenesis factors for tRFs remain elusive, designating tRFs as the molecules whose terminal structures have not yet been well defined. The tRFs that function as miRNAs or piRNAs by binding to AGO or PIWI proteins [32,43,44] should contain the 5′-P/3′-OH or 5′-P/2′-*O*-Me ends, respectively. tRFs further have a variety of functions, such as regulating gene expression via modulation of mRNA stability or translation, preventing cell apoptosis, and promoting virus infection, and their dysregulations are involved in various diseases [31,33,34,50].

### 2.4. Other sncRNAs

As in the case of tRFs, sdRNAs, the sncRNAs derived from small nucleolar RNAs (snoRNAs), have been reported to function as miRNAs by binding to AGO proteins and silencing the expression of target mRNAs [51,52,53]. While one of the sdRNAs has been reported to require Dicer, but not Drosha, for its production, not all sdRNAs are generated in a Dicer-dependent manner [51,53], suggesting that there are multiple biogenesis pathways for sdRNAs. Although their biogenesis pathways remain to be elucidated, these AGO-incorporated sdRNAs are expected to possess the 5′-P/3′-OH ends.

Ribosomal RNAs (rRNAs) and mRNAs are also utilized as substrates for sncRNAs [46,49,54,55,56,57,58]. While some of the rRNA- and mRNA-derived sncRNAs have been shown to function as miRNAs and are involved in gene silencing [58,59,60], the biological roles of many rRNA-/mRNA-derived sncRNAs remain unclear. Standard small RNA-seq captures numerous rRNA-/mRNA-derived sncRNAs, indicating the presence of 5′-P/3′-OH ends in these molecules; however, this does not mean that the 5′-P/3′-OH ends are their primary terminal formations. Indeed, many rRNA-/mRNA-derived sncRNAs have been identified as 2′,3′-cP-containing molecules [46,47]. Both rRNA- and mRNA-derived sncRNAs with the 2′,3′-cP end are mainly generated by specific cleavages between pyrimidine and adenosine [46,47]. The RNases responsible for the specific cleavages for the generation of 2′,3′-cP-containing molecules or for the biogenesis of other rRNA-/mRNA-derived sncRNAs have not been identified yet. The production of rRNA-derived sncRNAs seems to be largely Dicer- and Drosha-independent [49]. Because rRNAs and mRNAs undergo constitutive turnover, it is difficult to distinguish random degradation products from biologically significant sncRNAs. However, as in the case of tRFs, expression profiles of rRNA-derived sncRNAs are dependent on a person’s sex and population origin [61], and the expression of rRNA-/mRNA-derived 2′,3′-cP-containing sncRNAs are upregulated upon oxidative stress [47] and downregulated through aging [46]. Further research is needed to clarify biogenesis mechanisms and functionalities of these sncRNAs.

## 3. RNA-seq Methods Targeting sncRNAs with Specific Terminal Structures

The representative RNA-seq workflow includes: (1) extraction/purification of RNAs from cells/tissues, (2) construction of cDNA library, (3) next generation sequencing, and (4) bioinformatics analysis. Various strategies have been developed, mainly in the second step, to target specific sncRNAs with different terminal structures as follows.

### 3.1. Targeting sncRNAs with the 5′-P/3′-OH End

The standard method of cDNA library construction for sncRNAs utilizes adaptor (AD) ligations to the both 5′- and 3′-ends of sncRNAs. The ligated 5′- and 3′-ADs provide uniform hybridization sites for the primers in subsequent reverse transcription (RT) and PCR amplification. Commercially available kits for this standard method have been previously summarized [62]. Generally, a pre-adenylated 3′-AD containing a 5′,5′-adenyl pyrophosphoryl moiety is first ligated to the 3′-OH ends of sncRNAs by a truncated version of T4 RNA ligase 2, followed by 5′-AD ligation to the 5′-P end using T4 RNA ligase 1. The utilization of pre-adenylated 3′-AD prevents RNA self-ligation and concatenation, as the ligation reaction can be carried out in the absence of ATP. This standard RNA-seq method efficiently captures the sncRNAs with the 5′-P and 3′-OH ends that are required for the 5′- and 3′-AD ligations, respectively. The sncRNAs with other terminal formations cannot be ligated to the ADs and thus are not amplified by subsequent cDNA amplification steps, making these molecules invisible in standard RNA-seq data (Figure 1C).

### 3.2. Targeting sncRNAs with the 5′-OH/3′-OH End as Well as the 5′-P/3′-OH End

Circularizing AD-ligated RNA molecules is an alternative approach to AD ligation-based protocol [63]. The method uses a single 5′- and 3′-chimeric AD (pre-adenylated), which is ligated to the 3′-OH end of sncRNAs. After the treatment with T4 polynucleotide kinase (T4 PNK) to form the 5′-P and 3′-OH ends, the RNA-AD ligation products are subjected to self-ligation (circularization) using T4 RNA ligase 1, followed by cDNA amplification and sequencing. The advantage of this method includes more efficient intramolecular ligation compared to intermolecular ligation employed in standard RNA-seq. Because of the presence of a T4 PNK treatment step that phosphorylates the 5′-end of sncRNAs for circularization, 5′-OH-containing sncRNAs, as well as those with a 5′-P, are ligated and sequenced by this method, while the 3′-OH end is required for ligation to the chimeric AD. Therefore, this method captures the sncRNAs with both the 5′-P/3′-OH and 5′-OH/3′-OH ends (Figure 1C).

While the above two methods are based on AD ligation, there is a ligation-free sncRNA sequencing method that uses polyadenylation and template switching [64]. In this method, poly (A) polymerase adds poly (A) tail to the 3′-OH ends of RNAs, followed by RT using Moloney murine leukemia virus (MMLV) reverse transcriptase (RTase) and an oligo d(T) primer containing additional 3′-AD sequences. In this RT reaction, the MMLV RTase adds three to five deoxycytidines to the 3′-end of the produced cDNAs in a template-independent manner. The deoxycytidine-stretch serves as a priming site of template switching oligo (also serves as a forward PCR primer), and the switched templates are amplified by PCR. Because the 3′-OH ends of sncRNAs is required for the poly (A) addition by poly (A) polymerase, this method only captures the sncRNAs with the 3′-OH ends (i.e., those with the 5′-P/3′-OH and 5′-OH/3′-OH ends), but cannot capture those with a 3′-P or a 2′,3′-cP end (Figure 1C).

### 3.3. Targeting sncRNAs with the 2′-O-Me End

The above methods cannot efficiently amplify sncRNAs with the 2′-*O*-Me end, because the 3′-AD ligation or poly (A) addition to the 2′-*O*-Me end of RNAs are significantly inefficient [65,66,67,68]. Thus, when animal germline sncRNAs are subjected to standard RNA-seq, for example, miRNAs (containing the 5′-P/3′-OH end) will be efficiently captured, but generally more abundant piRNAs (containing the 5′-P/2′-*O*-Me end) will be significantly underrepresented in standard RNA-seq data. To overcome this issue and focus on the 2′-*O*-Me-containing sncRNAs, periodate oxidation has been used to enrich the 2′-*O*-Me-containing sncRNAs as substrates of 3′-AD ligation. Treatment of RNA fraction with sodium periodate (NaIO_4_) disrupts the 3′-OH ends of RNAs by transforming 2′,3′-cis diol moiety into 2′,3′-dialdehyde, which is no longer available for 3′-AD ligation. Therefore, the periodate treatment prior to AD ligations makes 2′-*O*-Me-containing RNAs sole RNA substrates for 3′-AD ligation. Even if the 3′-AD ligation to the 2′-*O*-Me end is inefficient, the enrichment step enables amplifying and sequencing 2′-*O*-Me-containing RNAs as major species (Figure 1C). The combination of periodate oxidation and RNA-seq has been used for the sequencings of 2′-*O*-Me-containing RNAs, mainly for animal piRNAs [44,69,70].

### 3.4. Targeting sncRNAs with the 2′,3′-cP End

Because 3′-AD cannot be ligated to the 2′,3′-cP end of RNAs, standard RNA-seq cannot capture 2′,3′-cP-containing RNAs. Instead, specific sequencing of sncRNAs with the 2′,3′-cP end can be achieved by cP-RNA-seq [37,71], which takes advantage of the distinct properties of T4 PNK and a phosphatase, such as calf intestinal phosphatase (CIP). While T4 PNK has 3′-terminal phosphatase activity that removes both 3′-P and 2′,3′-cP, the phosphatase activity of CIP hydrolyzes only 3′-P, but not 2′,3′-cP. In cP-RNA-seq, sncRNA fraction is first treated with CIP (convert the 3′-P end to the 3′-OH end) and then subjected to sodium periodate oxidation (disrupt the 3′-OH end). Unlike 3′-P and 3′-OH-containing RNAs, the 2′,3′-cP-containing RNAs survive from the 3′-end disruption. Consequently, after T4 PNK treatment (with ATP), which forms the 5′-P/3′-OH ends, the 2′,3′-cP-containing RNAs become the primary species for subsequent AD ligation and cDNA amplification steps, leading to their selective sequencing [37,71] (Figure 1C). As the 2′-*O*-Me end is resistant to periodate oxidation as described above, the 2′-*O*-Me-containing RNAs, as well as the 2′,3′-cP-containing RNAs, survive from the 3′-end disruption by periodate oxidation upon CIP treatment. However, the population of the 2′-*O*-Me-containing RNAs in cP-RNA-seq data is expected to be negligibly minor due to inefficient ligation of 3′-AD to the 2′-*O*-Me end. The cP-RNA-seq has been used to sequence 2′,3′-cP-containing tRNA halves in cancer cells [37,47], immune cells [38], and germ cells [43,44]. The first genome-wide identification of 2′,3′-cP-containing sncRNAs revealed numerous mRNA- and rRNA-derived 2′,3′-cP-containing sncRNAs, as well as tRNA halves, in various mouse tissues [46]. The development of P-cP-RNA-seq, a modified version of cP-RNA-seq that can specifically sequence sncRNAs with the 5′-P ‘and’ 2′,3′-cP ends, clarified the role of 2′,3′-cP-containing sncRNAs as direct piRNA precursors [44].

As an alternative method to selectively capture 2′,3′-cP-containing sncRNAs, *Arabidopsis thaliana* tRNA ligase (AtRNL) has been used [72,73]. Unlike the commonly used T4 RNA ligases 1 and 2, which ligate the 5′-P and 3′-OH ends of RNAs, the AtRNL can specifically ligate the 5′-OH and 2′,3′-cP ends [72]. Therefore, when AtRNL is used for the ligation reaction of 3′-AD with the 5′-OH end, 2′,3′-cP-containing sncRNAs become sole ligation substrates, leading to specific 3′-AD ligation to the RNAs (Figure 1C). The AtRNL ligation forms 2′-P at the ligation site, which should be removed by CIP or 2′-phosphotransferase such as *Saccharomyces cerevisiae* Tpt1 for efficient RT [72]. The sequencing of AtRNL-ligated RNAs has successfully identified 2′,3′-cP-containing sncRNAs such as U6 snRNA, tRNA halves, and 5′-cleavage products of self-cleaving ribozymes [72,73].

### 3.5. Targeting sncRNAs with the 5′-OH End

Standard RNA-seq cannot capture 5′-OH-containing RNAs because 5′-AD cannot be ligated to the 5′-OH ends of RNAs. Instead, specific sequencing of sncRNAs with the 5′-OH ends can be achieved by 5′-hydroxyl RNA-seq [74]. The method uses *Escherichia coli* RtcB RNA ligase, which can specifically ligate 3′-P-containing 5′-AD to the 5′-OH ends of RNAs, leading to specific amplification and sequencing of 5′-OH-containing RNAs (Figure 1C). The 5′-hydroxyl RNA-seq successfully captured numerous 5′-OH-containing mRNA-derived fragments that are likely to be generated by co-translational mRNA decay [74].

### 3.6. Targeting sncRNAs with All Terminal Phosphate States

In addition to targeting the specific terminal phosphate states as described above, broader range sncRNAs with all terminal phosphate states can be sequenced by Phospho-RNA-seq [75] in which RNA samples are treated with T4 PNK. Because T4 PNK possesses both 5′-phosphorylation (which converts 5′-OH to 5′-P in the presence of ATP) and 3′-dephosphorylation (which converts 3′-P/2′,3′-cP to 3′-OH) activities, the T4 PNK treatment converts the terminal phosphate states of all RNA species to the 5′-P/3′-OH-ends that are suitable for 5′-/3′-AD ligations in subsequent standard RNA-seq procedures (Figure 1C). This method comprehensively clarified the profiles of extracellular sncRNAs in human plasma samples and revealed tissue specific signatures in the profiles [75]. The T4 PNK treatment should be utilized to capture the whole picture of extracellular sncRNAs, as the majority of extracellular sncRNAs contain the 3′-P or 2′,3′-cP end [38,75] uncaptured by standard RNA-seq.

Not only terminal structures, but also internal post-transcriptional modifications of RNA molecules, can greatly affect sequencing efficiency. Some modifications block Watson–Crick base pairings and thus can interfere with RT, making the modified RNA molecules underrepresented in RNA-seq data. To overcome this issue, sequencing procedures for tRNAs (the most heavily-modified RNAs) and their fragments, such as DM-tRNA-seq [76] and AlkB-facilitated RNA methylation sequencing (ARM-seq) [77], include treatment of RNA fraction with *Escherichia coli* AlkB demethylase. The wild-type or engineered AlkB [76,78] can remove methylations from N^1^-methyladenosine (m^1^A), N^3^-methylcytidine (m^3^C), and N^1^-methylguanosine (m^1^G) residues, improving the efficiency of RT-PCR for the modified RNAs by erasing these RT-impairing modifications. Panoramic RNA display by overcoming RNA modification aborted sequencing (PANDORA-seq) combined the AlkB and T4-PNK treatments to fully capture sncRNAs [79]. Cap-Clip, T4 PNK, and AlkB/AlkB(D135S)-facilitated small ncRNA sequencing (CPA-seq) employed a treatment with Cap-Clip acid pyrophosphatase, as well as AlkB and T4 PNK treatments, to further capture sncRNAs with 5′-m^7^G cap structure and 5′-triphosphates [80]. Although still imperfect because AlkB cannot erase all RT-blocking modifications such as *N*^2^,*N*^2^-dimethylguanosine (m^2^_2_G), and because the biases generated by additional enzymatic treatments remain to be assessed in detail, the recent attempts to fully capture sncRNAs have shed light on previously unrecognized and uncharacterized sncRNAs.

## 4. Future Perspectives

Although the RNA-seq of T4 PNK-treated samples has an ability to capture broader sncRNA species compared to standard RNA-seq without any pre-treatment, it does not necessarily mean that the T4 PNK pretreatment of RNA is always required in all experiments. The information of terminal phosphates and modifications on RNA molecules are essential to understand the biogenesis mechanism and molecular function of the RNAs. T4 PNK treatment erases the terminal phosphate information of RNA molecules in sequencing data. It is also a possibility that the increased amounts and variations of the sncRNAs in the T4 PNK-treated RNA sequence data could mask the expression profiles of RNAs with relatively low abundance. For example, when the main research focus is on miRNAs, standard RNA-seq could be a better method than RNA-seq with T4 PNK treatment, because T4 PNK treatment vastly increases the abundance of non-miRNA reads (e.g., rRNA-/tRNA-/mRNA-derived sncRNAs), which could lessen the depths of the miRNA reads. These considerations speak to the importance of understanding the biogenesis pathways and resultant properties of each sncRNA. Although increasing accumulation of sequencing data from standard and modified RNA-seq has identified greater numbers of sncRNA species, the biogenesis pathways and responsible RNases remain unknown for large parts of the sncRNAs. Without knowing the biogenesis enzymes and their generating terminal phosphate states of the targeted sncRNAs, it is difficult to determine an appropriate sequencing method for the RNAs and accurately interpret RNA-seq data. More importantly, to advance our understanding of the expanding realm of sncRNA, it is imperative to distinguish functional sncRNAs from random degradation products. Further research to accumulate the data of previously “hidden” sncRNA from the above-described modified versions of RNA-seq to capture specific sncRNAs or whole sncRNAome will not only reveal expressional regulation of wider repertoires of sncRNAs, but also help their functional characterization, potentially clarifying substantially greater biological significance of sncRNAs.

## References

[1] Cerami E., Gao J., Dogrusoz U., Gross B.E., Sumer S.O., Aksoy B.A., Jacobsen A., Byrne C.J., Heuer M.L., Larsson E. (2012). The cBio cancer genomics portal: An open platform for exploring multidimensional cancer genomics data. Cancer Discov..

[2] Gao J., Aksoy B.A., Dogrusoz U., Dresdner G., Gross B., Sumer S.O., Sun Y., Jacobsen A., Sinha R., Larsson E. (2013). Integrative analysis of complex cancer genomics and clinical profiles using the cBioPortal. Sci. Signal..

[3] Tate J.G., Bamford S., Jubb H.C., Sondka Z., Beare D.M., Bindal N., Boutselakis H., Cole C.G., Creatore C., Dawson E. (2019). COSMIC: The Catalogue Of Somatic Mutations In Cancer. Nucleic Acids Res..

[4] Lee R.C., Feinbaum R.L., Ambros V. (1993). The C. elegans heterochronic gene lin-4 encodes small RNAs with antisense complementarity to lin-14. Cell.

[5] Wightman B., Ha I., Ruvkun G. (1993). Posttranscriptional regulation of the heterochronic gene lin-14 by lin-4 mediates temporal pattern formation in C. elegans. Cell.

[6] Bartel D.P. (2004). MicroRNAs: Genomics, biogenesis, mechanism, and function. Cell.

[7] Pillai R.S., Bhattacharyya S.N., Filipowicz W. (2007). Repression of protein synthesis by miRNAs: How many mechanisms?. Trends Cell Biol..

[8] Bartel D.P. (2009). MicroRNAs: Target recognition and regulatory functions. Cell.

[9] Jonas S., Izaurralde E. (2015). Towards a molecular understanding of microRNA-mediated gene silencing. Nat. Rev. Genet..

[10] MacRae I.J., Doudna J.A. (2007). Ribonuclease revisited: Structural insights into ribonuclease III family enzymes. Curr. Opin. Struct. Biol..

[11] Kim V.N. (2005). MicroRNA biogenesis: Coordinated cropping and dicing. Nat. Rev. Mol. Cell Biol..

[12] Ha M., Kim V.N. (2014). Regulation of microRNA biogenesis. Nat. Rev. Mol. Cell Biol..

[13] Yu B., Yang Z., Li J., Minakhina S., Yang M., Padgett R.W., Steward R., Chen X. (2005). Methylation as a crucial step in plant microRNA biogenesis. Science.

[14] Borges F., Martienssen R.A. (2015). The expanding world of small RNAs in plants. Nat. Rev. Mol. Cell Biol..

[15] Ghildiyal M., Zamore P.D. (2009). Small silencing RNAs: An expanding universe. Nat. Rev. Genet..

[16] Piatek M.J., Werner A. (2014). Endogenous siRNAs: Regulators of internal affairs. Biochem. Soc. Trans..

[17] Ji L., Chen X. (2012). Regulation of small RNA stability: Methylation and beyond. Cell Res..

[18] Kim V.N., Han J., Siomi M.C. (2009). Biogenesis of small RNAs in animals. Nat. Rev. Mol. Cell Biol..

[19] Ozata D.M., Gainetdinov I., Zoch A., O’Carroll D., Zamore P.D. (2019). PIWI-interacting RNAs: Small RNAs with big functions. Nat. Rev. Genet..

[20] Czech B., Munafo M., Ciabrelli F., Eastwood E.L., Fabry M.H., Kneuss E., Hannon G.J. (2018). piRNA-Guided Genome Defense: From Biogenesis to Silencing. Annu. Rev. Genet..

[21] Ipsaro J.J., Haase A.D., Knott S.R., Joshua-Tor L., Hannon G.J. (2012). The structural biochemistry of Zucchini implicates it as a nuclease in piRNA biogenesis. Nature.

[22] Nishimasu H., Ishizu H., Saito K., Fukuhara S., Kamatani M.K., Bonnefond L., Matsumoto N., Nishizawa T., Nakanaga K., Aoki J. (2012). Structure and function of Zucchini endoribonuclease in piRNA biogenesis. Nature.

[23] Brennecke J., Aravin A.A., Stark A., Dus M., Kellis M., Sachidanandam R., Hannon G.J. (2007). Discrete small RNA-generating loci as master regulators of transposon activity in Drosophila. Cell.

[24] Gunawardane L.S., Saito K., Nishida K.M., Miyoshi K., Kawamura Y., Nagami T., Siomi H., Siomi M.C. (2007). A slicer-mediated mechanism for repeat-associated siRNA 5’ end formation in Drosophila. Science.

[25] Izumi N., Shoji K., Sakaguchi Y., Honda S., Kirino Y., Suzuki T., Katsuma S., Tomari Y. (2016). Identification and Functional Analysis of the Pre-piRNA 3’ Trimmer in Silkworms. Cell.

[26] Horwich M.D., Li C., Matranga C., Vagin V., Farley G., Wang P., Zamore P.D. (2007). The Drosophila RNA methyltransferase, DmHen1, modifies germline piRNAs and single-stranded siRNAs in RISC. Curr. Biol..

[27] Saito K., Sakaguchi Y., Suzuki T., Suzuki T., Siomi H., Siomi M.C. (2007). Pimet, the Drosophila homolog of HEN1, mediates 2’-O-methylation of Piwi- interacting RNAs at their 3’ ends. Genes Dev..

[28] Kirino Y., Mourelatos Z. (2007). The mouse homolog of HEN1 is a potential methylase for Piwi-interacting RNAs. RNA.

[29] Kirino Y., Mourelatos Z. (2007). Mouse Piwi-interacting RNAs are 2’-O-methylated at their 3’ termini. Nat. Struct. Mol. Biol..

[30] Ohara T., Sakaguchi Y., Suzuki T., Ueda H., Miyauchi K. (2007). The 3’ termini of mouse Piwi-interacting RNAs are 2’-O-methylated. Nat. Struct. Mol. Biol..

[31] Kumar P., Kuscu C., Dutta A. (2016). Biogenesis and Function of Transfer RNA-Related Fragments (tRFs). Trends Biochem. Sci..

[32] Shigematsu M., Kirino Y. (2015). tRNA-Derived Short Non-coding RNA as Interacting Partners of Argonaute Proteins. Gene Regul. Syst. Biol..

[33] Anderson P., Ivanov P. (2014). tRNA fragments in human health and disease. FEBS Lett..

[34] Magee R., Rigoutsos I. (2020). On the expanding roles of tRNA fragments in modulating cell behavior. Nucleic Acids Res..

[35] Yamasaki S., Ivanov P., Hu G.F., Anderson P. (2009). Angiogenin cleaves tRNA and promotes stress-induced translational repression. J. Cell Biol..

[36] Fu H., Feng J., Liu Q., Sun F., Tie Y., Zhu J., Xing R., Sun Z., Zheng X. (2009). Stress induces tRNA cleavage by angiogenin in mammalian cells. FEBS Lett..

[37] Honda S., Loher P., Shigematsu M., Palazzo J.P., Suzuki R., Imoto I., Rigoutsos I., Kirino Y. (2015). Sex hormone-dependent tRNA halves enhance cell proliferation in breast and prostate cancers. Proc. Natl. Acad. Sci. USA.

[38] Pawar K., Shigematsu M., Sharbati S., Kirino Y. (2020). Infection-induced 5’-half molecules of tRNAHisGUG activate Toll-like receptor 7. PLoS Biol..

[39] Kawamura T., Shigematsu M., Kirino Y. In vitro production and multiplex quantification of 2’,3’-cyclic phosphate-containing 5’-tRNA half molecules. Methods.

[40] Emara M.M., Ivanov P., Hickman T., Dawra N., Tisdale S., Kedersha N., Hu G.F., Anderson P. (2010). Angiogenin-induced tRNA-derived stress-induced RNAs promote stress-induced stress granule assembly. J. Biol. Chem..

[41] Ivanov P., Emara M.M., Villén J., Gygi S.P., Anderson P. (2011). Angiogenin-Induced tRNA Fragments Inhibit Translation Initiation. Mol. Cell.

[42] Fricker R., Brogli R., Luidalepp H., Wyss L., Fasnacht M., Joss O., Zywicki M., Helm M., Schneider A., Cristodero M. (2019). A tRNA half modulates translation as stress response in Trypanosoma brucei. Nat. Commun..

[43] Honda S., Kawamura T., Loher P., Morichika K., Rigoutsos I., Kirino Y. (2017). The biogenesis pathway of tRNA-derived piRNAs in Bombyx germ cells. Nucleic Acids Res..

[44] Shigematsu M., Kawamura T., Morichika K., Izumi N., Kiuchi T., Honda S., Pliatsika V., Matsubara R., Rigoutsos I., Katsuma S. (2021). RNase kappa promotes robust piRNA production by generating 2’,3’-cyclic phosphate-containing precursors. Nat. Commun..

[45] Shapiro R., Riordan J.F., Vallee B.L. (1986). Characteristic ribonucleolytic activity of human angiogenin. Biochemistry.

[46] Shigematsu M., Morichika K., Kawamura T., Honda S., Kirino Y. (2019). Genome-wide identification of short 2’,3’-cyclic phosphate-containing RNAs and their regulation in aging. PLoS Genet..

[47] Shigematsu M., Kirino Y. (2020). Oxidative stress enhances the expression of 2’,3’-cyclic phosphate-containing RNAs. RNA Biol..

[48] Haussecker D., Huang Y., Lau A., Parameswaran P., Fire A.Z., Kay M.A. (2010). Human tRNA-derived small RNAs in the global regulation of RNA silencing. RNA.

[49] Li Z., Ender C., Meister G., Moore P.S., Chang Y., John B. (2012). Extensive terminal and asymmetric processing of small RNAs from rRNAs, snoRNAs, snRNAs, and tRNAs. Nucleic Acids Res..

[50] Yu X., Xie Y., Zhang S., Song X., Xiao B., Yan Z. (2021). tRNA-derived fragments: Mechanisms underlying their regulation of gene expression and potential applications as therapeutic targets in cancers and virus infections. Theranostics.

[51] Taft R.J., Glazov E.A., Lassmann T., Hayashizaki Y., Carninci P., Mattick J.S. (2009). Small RNAs derived from snoRNAs. RNA.

[52] Ender C., Krek A., Friedlander M.R., Beitzinger M., Weinmann L., Chen W., Pfeffer S., Rajewsky N., Meister G. (2008). A human snoRNA with microRNA-like functions. Mol. Cell.

[53] Wajahat M., Bracken C.P., Orang A. (2021). Emerging Functions for snoRNAs and snoRNA-Derived Fragments. Int. J. Mol. Sci..

[54] Lambert M., Benmoussa A., Provost P. (2019). Small Non-Coding RNAs Derived From Eukaryotic Ribosomal RNA. Noncoding RNA.

[55] Jackowiak P., Hojka-Osinska A., Philips A., Zmienko A., Budzko L., Maillard P., Budkowska A., Figlerowicz M. (2017). Small RNA fragments derived from multiple RNA classes—The missing element of multi-omics characteristics of the hepatitis C virus cell culture model. BMC Genom..

[56] Burroughs A.M., Ando Y., de Hoon M.J., Tomaru Y., Suzuki H., Hayashizaki Y., Daub C.O. (2011). Deep-sequencing of human Argonaute-associated small RNAs provides insight into miRNA sorting and reveals Argonaute association with RNA fragments of diverse origin. RNA Biol..

[57] Pircher A., Bakowska-Zywicka K., Schneider L., Zywicki M., Polacek N. (2014). An mRNA-derived noncoding RNA targets and regulates the ribosome. Mol. Cell.

[58] McCue A.D., Panda K., Nuthikattu S., Choudury S.G., Thomas E.N., Slotkin R.K. (2015). ARGONAUTE 6 bridges transposable element mRNA-derived siRNAs to the establishment of DNA methylation. EMBO J..

[59] Castellano L., Stebbing J. (2013). Deep sequencing of small RNAs identifies canonical and non-canonical miRNA and endogenous siRNAs in mammalian somatic tissues. Nucleic Acids Res..

[60] Chak L.L., Mohammed J., Lai E.C., Tucker-Kellogg G., Okamura K. (2015). A deeply conserved, noncanonical miRNA hosted by ribosomal DNA. RNA.

[61] Cherlin T., Magee R., Jing Y., Pliatsika V., Loher P., Rigoutsos I. (2020). Ribosomal RNA fragmentation into short RNAs (rRFs) is modulated in a sex- and population of origin-specific manner. BMC Biol..

[62] Benesova S., Kubista M., Valihrach L. (2021). Small RNA-Sequencing: Approaches and Considerations for miRNA Analysis. Diagnostics.

[63] Barberan-Soler S., Vo J.M., Hogans R.E., Dallas A., Johnston B.H., Kazakov S.A. (2018). Decreasing miRNA sequencing bias using a single adapter and circularization approach. Genome Biol..

[64] Berezikov E., Cuppen E., Plasterk R.H. (2006). Approaches to microRNA discovery. Nat. Genet..

[65] Dard-Dascot C., Naquin D., d’Aubenton-Carafa Y., Alix K., Thermes C., van Dijk E. (2018). Systematic comparison of small RNA library preparation protocols for next-generation sequencing. BMC Genom..

[66] Munafo D.B., Robb G.B. (2010). Optimization of enzymatic reaction conditions for generating representative pools of cDNA from small RNA. RNA.

[67] Yang Z., Ebright Y.W., Yu B., Chen X. (2006). HEN1 recognizes 21-24 nt small RNA duplexes and deposits a methyl group onto the 2’ OH of the 3’ terminal nucleotide. Nucleic Acids Res..

[68] Wang N., Qu S., Sun W., Zeng Z., Liang H., Zhang C.Y., Chen X., Zen K. (2018). Direct quantification of 3’ terminal 2’-O-methylation of small RNAs by RT-qPCR. RNA.

[69] Williams Z., Morozov P., Mihailovic A., Lin C., Puvvula P.K., Juranek S., Rosenwaks Z., Tuschl T. (2015). Discovery and Characterization of piRNAs in the Human Fetal Ovary. Cell Rep..

[70] Sarkies P., Selkirk M.E., Jones J.T., Blok V., Boothby T., Goldstein B., Hanelt B., Ardila-Garcia A., Fast N.M., Schiffer P.M. (2015). Ancient and novel small RNA pathways compensate for the loss of piRNAs in multiple independent nematode lineages. PLoS Biol..

[71] Honda S., Morichika K., Kirino Y. (2016). Selective amplification and sequencing of cyclic phosphate-containing RNAs by the cP-RNA-seq method. Nat. Protoc..

[72] Schutz K., Hesselberth J.R., Fields S. (2010). Capture and sequence analysis of RNAs with terminal 2’,3’-cyclic phosphates. RNA.

[73] Olzog V.J., Gartner C., Stadler P.F., Fallmann J., Weinberg C.E. (2021). cyPhyRNA-seq: A genome-scale RNA-seq method to detect active self-cleaving ribozymes by capturing RNAs with 2’,3’ cyclic phosphates and 5’ hydroxyl ends. RNA Biol..

[74] Peach S.E., York K., Hesselberth J.R. (2015). Global analysis of RNA cleavage by 5’-hydroxyl RNA sequencing. Nucleic Acids Res..

[75] Giraldez M.D., Spengler R.M., Etheridge A., Goicochea A.J., Tuck M., Choi S.W., Galas D.J., Tewari M. (2019). Phospho-RNA-seq: A modified small RNA-seq method that reveals circulating mRNA and lncRNA fragments as potential biomarkers in human plasma. EMBO J..

[76] Zheng G., Qin Y., Clark W.C., Dai Q., Yi C., He C., Lambowitz A.M., Pan T. (2015). Efficient and quantitative high-throughput tRNA sequencing. Nat. Methods.

[77] Cozen A.E., Quartley E., Holmes A.D., Hrabeta-Robinson E., Phizicky E.M., Lowe T.M. (2015). ARM-seq: AlkB-facilitated RNA methylation sequencing reveals a complex landscape of modified tRNA fragments. Nat. Methods.

[78] Wang Y., Katanski C.D., Watkins C., Pan J.N., Dai Q., Jiang Z., Pan T. (2021). A high-throughput screening method for evolving a demethylase enzyme with improved and new functionalities. Nucleic Acids Res..

[79] Shi J., Zhang Y., Tan D., Zhang X., Yan M., Zhang Y., Franklin R., Shahbazi M., Mackinlay K., Liu S. (2021). PANDORA-seq expands the repertoire of regulatory small RNAs by overcoming RNA modifications. Nat. Cell Biol..

[80] Wang H., Huang R., Li L., Zhu J., Li Z., Peng C., Zhuang X., Lin H., Shi S., Huang P. (2021). CPA-seq reveals small ncRNAs with methylated nucleosides and diverse termini. Cell Discov..

